# A Treatise on the Decline of Manhood, Etc.

**Published:** 1881-09

**Authors:** 


A TREATISE ON THE DECLINE OF MANHOOD; ITS CAUSES, AND THE BEST MEANS OF PREVENTING THEIR EFFECTS AND BRINGING ABOUT A RESTORATION TO HEALTH. By *A. E. Small, A. M., M. D.* Chicago: Duncan Brothers. 1881.

This little book of 102 pages is, according to the preface, "an attempt to supply a want in the literature of homœopathy." This we are not quite ready to grant that it does. The treatment is not sufficiently comprehensive. The remedies given are only a few that have been successful in the experience of the author. The differential indications are vague and general. The treatment is too suggestive of the empiricism of the old school. This empiricism has already made considerable inroads into the avowedly homœopathic literature, and it is not desirable that it should be encouraged. Dr. Small should, therefore, give a more extended symtomatology, like that in Bell's book on Diarrhœa; his work would then become a hand-book for daily use. On page 42, "*Arum muriaticum*" should read *Aurum muriaticum.* Do not the symptoms enumerated at foot of page 57 indicate *Cantharis* rather than *Tartar Em.?*

